# 3D-printer visualization of neuron models

**DOI:** 10.3389/fninf.2015.00018

**Published:** 2015-06-30

**Authors:** Robert A. McDougal, Gordon M. Shepherd

**Affiliations:** Department of Neurobiology, Yale UniversityNew Haven, CT, USA

**Keywords:** database, visualization, 3d printing, morphology, modeling

## Abstract

Neurons come in a wide variety of shapes and sizes. In a quest to understand this neuronal diversity, researchers have three-dimensionally traced tens of thousands of neurons; many of these tracings are freely available through online repositories like NeuroMorpho.Org and ModelDB. Tracings can be visualized on the computer screen, used for statistical analysis of the properties of different cell types, used to simulate neuronal behavior, and more. We introduce the use of 3D printing as a technique for visualizing traced morphologies. Our method for generating printable versions of a cell or group of cells is to expand dendrite and axon diameters and then to transform the tracing into a 3D object with a neuronal surface generating algorithm like Constructive Tessellated Neuronal Geometry (CTNG). We show that 3D printed cells can be readily examined, manipulated, and compared with other neurons to gain insight into both the biology and the reconstruction process. We share our printable models in a new database, 3DModelDB, and encourage others to do the same with cells that they generate using our code or other methods. To provide additional context, 3DModelDB provides a simulatable version of each cell, links to papers that use or describe it, and links to associated entries in other databases.

## 1. Introduction

The nervous system contains the most complex 3-dimensional structures of any organ in the body. This applies at all levels: the gross brain, the individual regions, the circuits within and between regions, and the neurons themselves. Visualizing these structures in their true 3-dimensional morphology is therefore a critical challenge in relating structure to function.

There has been slow but steady progress in the ability to visualize and study neuron morphologies. The Golgi method, developed in the late nineteenth century, was the first technique to allow distinguishing individual neurons with a microscope. Modern computer technology allows researchers to trace neurons in 3D from microscopy images to quantify the morphology (Glaser and Glaser, 1990; Al-Kofahi et al., 2002; Kaynig et al., 2015). This quantified morphology can be analyzed statistically or rendered on a computer screen. Because the renderings can be mathematically rotated, a 2 dimensional computer screen can be used to examine a neuron's 3 dimensional nature. However, independently traced cells cannot reveal the nature of connections in a local microcircuit because the overlapping dendritic trees will likely be incompatible. One current strategy for predicting microcircuit structure is to virtually grow the microcircuit together where each cell's morphology is based on statistical properties of traced cells (Donohue and Ascoli, 2008; Zubler and Douglas, 2009; Cuntz et al., 2010; Wolf et al., 2013; Migliore et al., 2014).

We introduce 3D printed neurons as a new and potentially valuable tool for visualizing morphologies of individual neurons and the connections between neurons in a microcircuit. This had not been previously attempted because of the extremely intricate and delicate nature of dendritic branching. Modern high-end 3D printers are capable of printing these structures albeit with some loss of detail about dendritic diameters. Although the necessary hardware for performing such printouts on site remains outside the budget of most labs including ours, commercial printing services now exist that can affordably print individual neurons. We report here our method for making the first neurons printable with this technology and a database for freely sharing printable neuron models. We give examples of how these physical 3D neurons can give insight into the 3D architecture of neurons and into the way neurons interact to form microcircuits. We note that printed cells also provide dramatic examples of the intricacy of neurons to aid in neuroscience education.

## 2. Materials and methods

### 2.1. Creating a model

**Figure 1:**
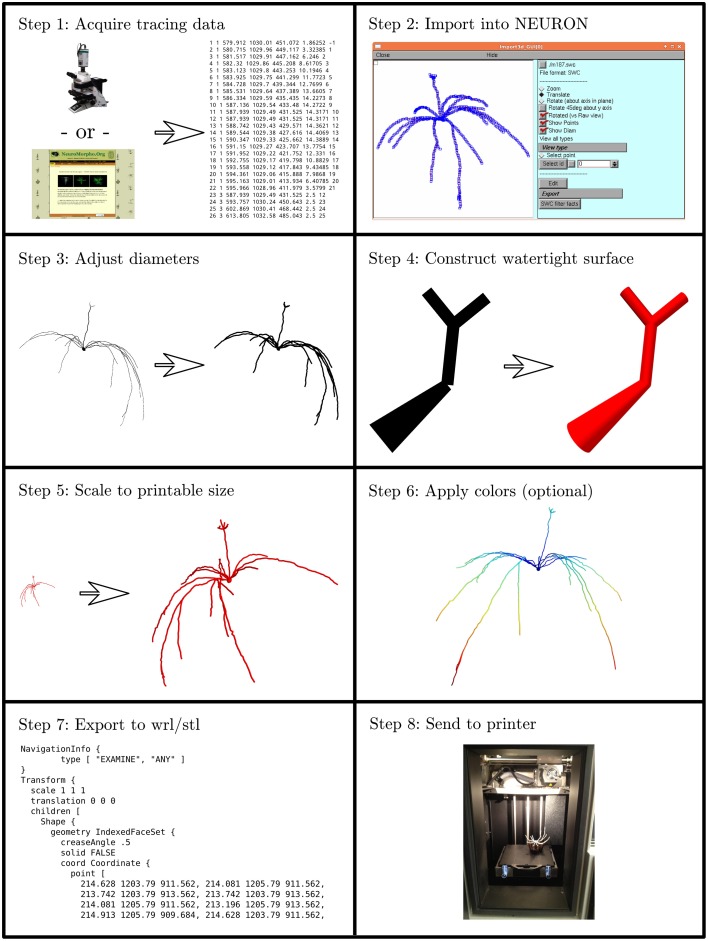
**The process of making a printable model, illustrated with the first neuron we printed, which is available in 3DModelDB as “m187.”** Morphology must first be acquired, either from tracing a cell, a computational model, or a database. The morphology is then loaded into NEURON which is used to manipulate diameters and sizes and to construct a watertight surface. At this stage, colors may optionally be applied. The surface is then exported to WRL or STL and then sent to a 3D printer. Step 8 shows the non-consumer grade FFF printer used for our first two printouts; after that, we switched to a commercial SLS printer.

We developed an eight step process to create 3D printable neuron models. We implemented this process in custom Python code which is available at http://modeldb.yale.edu/182785. This process is summarized in Figure 1 and described in detail below.

#### 2.1.1. Step 1: acquire tracing data

Our first step for making a 3D printable model was to acquire morphology data in a computer-representable form (Figure 1 Step 1). Our initial goal was to better understand the synthetic mitral cells in the computational model of Migliore et al. (2014), so our first tests used some of those cells and a microcircuit from that model. Other models have been made from Neurolucida (Glaser and Glaser, 1990) tracings, tracings in the SWC format (Cannon et al., 1998), and from ModelDB entries (Hines et al., 2004). We took the tracings from NeuroMorpho.Org (Ascoli et al., 2007). Tracings in Neurolucida form have a slight advantage, as that format optionally includes soma outlines which can be used to produce a more plausible looking soma.

#### 2.1.2. Step 2: import the data into NEURON

We then loaded the morphologies into NEURON (Hines and Carnevale, 1997) (Figure 1 Step 2). NEURON was used because of our familiarity with it, because our initial morphologies of interest were built in NEURON, and because it offers support for reading several morphology types. This support allowed us to write a single code for manipulating multiple formats. For those models not already in NEURON code, we used NEURON's Import 3D tool either interactively (Tools—Miscellaneous—Import 3D) or via a script. During the import process, NEURON sometimes identified issues with the reconstructions. For most issues, depending on the severity, we either rejected the reconstruction or accepted an automatically suggested fix. In the special case of dendrites that are detached from the soma, we extended the dendrite at a constant diameter to the centroid of the soma. We deviated from the NEURON automatic fix—repositioning the dendrite—because we considered the measured coordinates canonical and because in many cases (in particular with SWC files) the soma in the reconstruction data was simply represented via a sphere. For some models, we removed the axon immediately after importing the morphology.

#### 2.1.3. Step 3: adjust diameters

3D printers impose practical limits on the minimum thickness of wirey structures like neurites (dendrites and axons) to allow them to resist the pull of gravity and provide enough strength to keep the model intact. Unfortunately, for the printers we used, this minimum thickness is several times thicker than would be obtained by simply scaling up the biology. We thus iterated over all the non-soma sections in NEURON and enlarged their diameters while preserving the lengths (Figure 1 Step 3). In NEURON, the soma, like a dendrite, is represented internally by its centroid and a set of diameters. Enlarging the diameter alone would distort the soma shape. Instead, we applied a constant scale factor to both diameters and positions. The enlarged soma was then repositioned so that its center of mass was the same as that of the original soma.

We employed one of several strategies to adjust the dendrite diameters: most commonly, we imposed a fixed diameter for all dendrites. In cases where we wanted the apical dendrite or axon to stand out, we sometimes used a different fixed diameter for those regions. In still other cases, we applied a scale factor to axon or dendrite diameter or imposed a minimum diameter. Distinguishing between axon, dendrite, and soma is easy in NEURON because the Import 3D tool encodes this type of information from the source file into the section names.

#### 2.1.4. Step 4: construct watertight surface

We then transformed the representation from one based on points and diameters to a watertight one based on surface triangles. We used the constructive tessellated neuronal geometry (CTNG) of McDougal et al. (2013) (Figure 1 Step 4). There are other algorithms that also construct watertight surfaces, however CTNG has the advantage of being a local algorithm, and is thus able to handle interpenetrating surfaces caused by dendrites passing close to each other, either from the same cell or from different cells. Even in that case, CTNG is able to form a single unified surface. The discretization resolution for CTNG was chosen by trial-and-error to be small enough to resolve the rescaled dendrites but large enough to keep the total number of triangles generated small. The original source code for CTNG is available at http://modeldb.yale.edu/146950.

#### 2.1.5. Step 5: scale to printable size

We then scaled the triangles to their final printed size (Figure 1 Step 5). We originally did this by rendering them in Mayavi (Ramachandran and Varoquaux, 2011), exporting to WRL which is also known as VRML (Carey and Bell, 1997), opening them in MeshLab, and resizing them to a desired size. We later switched to multiplying the *x*, *y*, and *z* coordinates of the model by an appropriately chosen scale factor. For morphologies measured in microns and printer software expecting millimeters, multiplying the coordinates by 0.2 corresponds to a 200x magnification.

#### 2.1.6. Step 6: apply colors (optional)

Color may be applied at this step (Figure 1 Step 6). As CTNG does not preserve information about logical structure when constructing surfaces, if the color is to be chosen based on the logical structure, each surface triangle must be mapped back to the underlying structure. As NEURON conceptualizes each segment as a series of frusta, this mapping can be done by finding which frusta the centroid of the triangle is inside but inside by the least amount. If there is no such frusta, then the triangle is mapped to the segment with the frustum whose surface is the closest. Color information may then be assigned based on the segment (e.g., by segment name, by properties of the segment like voltages or conductances in the original unscaled NEURON model, etc.).

#### 2.1.7. Step 7: export to WRL or STL

Except in our first few prints—where this was done earlier—we then rendered our models with Mayavi (Ramachandran and Varoquaux, 2011) and saved them from Mayavi into WRL. When we printed on machines that required STL format instead, we opened the WRL in MeshLab (3D-CoForm project) and resaved it as STL. We note that STL does not preserve color information.

#### 2.1.8. Step 8: print the model

Our initial models were printed at Yale's Center for Engineering Innovation and Design (CEID) on a Stratasys Dimension Elite 3D printer, a Fused Filament Fabrication (FFF) printer (Figure 1 Step 8). This machine was chosen because it uses a soluble material to build the support structure to hold the dendrites in place while the neuron is being printed. After printing, the neuron with its support structure was placed in a bath which fully dissolved the supports. This first successful attempt was reported briefly in a news item by Kurzweil in 2013 (http://www.kurzweilai.net/first-3d-printed-model-of-a-neuron).

In an attempt to find a printing strategy more accessible for neuroscience labs including our own—the CEID only has one Dimension Elite machine that serves the entire university—we attempted to print on a MakerBot Replicator 2X, a consumer-grade FFF printer. Printing without supports created a flat printout as the dendrites collapsed while being printed. Printing with supports was rejected as too impractical because the supports would need to be manually removed without damaging the neuron's delicate structure.

We thus switched to printing our neuron models at a commercial provider using Selective Laser Sintering (SLS; reviewed in Kruth et al., 2003). SLS avoids support structure issues by printing in a basin filled with powder; this powder holds everything in place during printing, and it is easily cleaned off after printing. There are many such commercial providers available; we used Shapeways and typically printed in their White Strong and Flexible material. We also successfully used their service to print in black, in dyed plastic, and in metal.

### 2.2. Model sharing

We created a database, 3DModelDB (http://senselab.med.yale.edu/3dmodeldb) to share 3D printable neuron reconstructions. The backend of the database is implemented with EAV/CR (Nadkarni et al., 1999) and is now an integrated part of the SenseLab suite of neuroscience databases. In particular, it is connected with NeuronDB (for neuron properties) and with ModelDB (for computational neuroscience models). The dynamic web pages are rendered using ASP.NET's Razor view engine. Full text and attribute search are powered by a custom Python backend.

The frontend is powered by several JavaScript frameworks. The jQuery library (http://jquery.com) is required by many of the other libraries and is used to simplify the custom JavaScript. Bootstrap (http://getbootstrap.com) and jQuery UI (http://jqueryui.com) provide some of the graphical elements. Slimbox 2 (http://digitalia.be/software/slimbox2/) is used to frame large versions of photographs. Three.js (http://threejs.org) is used to control WebGL which powers an interactive 3D neuron viewer that works on any HTML5 capable browser; no plugins are required. We provide a static image of the neuron for older browsers.

## 3. Results

### 3.1. Neuron printability

Not every 3D model is printable. Researchers with their own printers may push the boundaries of their hardware as much as they want by risking print failure, but for those like ourselves using commercial printers, we are limited by what the service is willing to attempt to print.

When printing in their White Strong and Flexible material, our commercial provider imposes a minimum 1 mm thickness for wirey structures like dendrites and axons. The dendrites of our first test, a mitral cell, are on the order of a 2–3 microns in diameter but over a millimeter in length; magnifying the model around 500x to get the dendrites at the correct thickness would create dendrites longer than half a meter. This is bigger than their machines could physically handle, and even if it could, the long thin dendrites would likely be too flexible to retain their shape against the pull of gravity. This fact is what led to the decision to use unnaturally thick dendrites in the printouts. Due to the way CTNG works, the tessellated surface may be slightly thinner than described by the logical structure, so to avoid models being rejected for not meeting their guidelines, we found it more effective to submit models with 1.2 mm thick dendrites than to aim for their minimum.

Meeting the minimum thicknesses requested by the printer does not guarantee that it will print successfully. We submitted 51 distinct models meeting their requirements to our commercial provider for printing in their White Strong and Flexible material. One of these, NeuroMorpho.Org ID NMO_05958 (Brunjes and Kenerson, 2010), with a long apical and a heavy soma due to high (800x) magnification was rejected as “likely [to] break in the post production process.” Another, a basket cell, NeuroMorpho.Org ID NMO_10716 (Tukker et al., 2013), was printed and did break during post-production, presumably due to its large poorly supported basket. The other 49 models were able to be printed successfully, although 6 of those did not print successfully every time. The 6 models with an imperfect record were converted from: ModelDB model 114394 (Kole et al., 2008), ModelDB model 119266 (Markaki et al., 2005), ModelDB model 136026 (Djurisic et al., 2008), NeuroMorpho.Org model NMO_00607 (Cullheim et al., 1987), NeuroMorpho.Org model NMO_05518 (Radman et al., 2009), and NeuroMorpho.Org model NMO_00227 (Ishizuka et al., 1995). Each of these neurons features long, unbranched sections of neurites.

Requests for thicker than the minimum requested dendrites were not limited to that material. When we attempted to print glomerular cluster 37 from Migliore et al. (2014) in Full Color Sandstone, the guidelines called for 3 mm thick dendrites, but we were requested to thicken them further to 5 mm.

We encountered other difficulties when trying to print in metal. The commercial service we used does this by printing a wax mold and then filling it with metal. They advised us that it would be impractical to print one of our models this way because the metal would have to be added at several points as it would not flow freely through the mold.

### 3.2. Printable model database

We created 3DModelDB (http://senselab.med.yale.edu/3dmodeldb) as a place to share 3D printable models, both from our lab and elsewhere.

**Figure 2:**
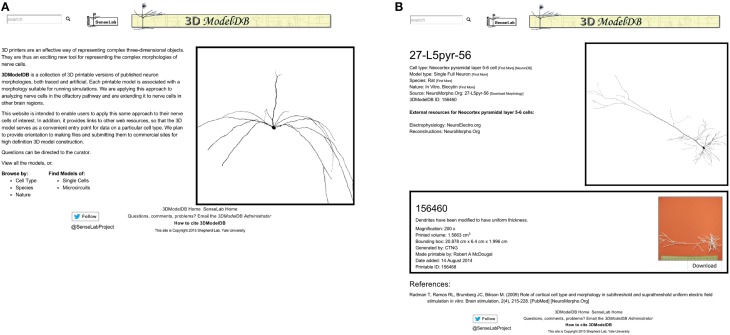
**(A)** The homepage offers links to browse by cell type, by species, and more. **(B)** This page for a specific pyramidal cell displays metadata about the cell, links to more information about the cell type in other databases, an interactive 3D viewer powered by WebGL, and in this case one—sometimes more—3D printable representation of the cell for downloading. This morphology is from Radman et al. (2009) via NeuroMorpho.Org.

The 3DModelDB homepage (Figure 2) offers links to view the full current list of printable cells or to browse by cell type, species, single cells vs. microcircuits, or tracing technique. The types of neurons initially in our database are listed in Table 1. The browser page lists cell name (chosen to match NeuroMorpho.Org for those cells that entered our database from there), species, cell type, whether a model is for a single cell or microcircuit, tracing technique, and number of printable versions for each cell. The results may be sorted by any of these categories. Alternatively, a model may be located by typing a model name or attribute value into the search box at the upper left of every 3DModelDB page.

**Table 1:** **Cell types in 3DModelDB as of February 26, 2015**.

Anterior olfactory nucleus pyramidal cell
Cerebellum purkinje cell
Dentate gyrus granule cell
Hippocampus CA1 pyramidal cell
Lumbar spinal cord projection neuron
Neocortex basket cell
Neocortex pyramidal layer 5-6 cell
Olfactory bulb mitral cell
Olfactory bulb tufted cell
Spinal cord motor neuron
Thalamus relay cell

Selecting a cell opens a webpage (Figure 2 right) that presents metadata about the cell, a link to a simulatable version of the cell, an interactive 3d image, and 3D printable versions of the cells.

The metadata includes cell type (Thalamus relay cell, Hippocampus CA1 pyramidal cell, etc.), model type (Single Full Neuron, Microcircuit), species (rat, cat, etc.), and nature (*in vivo* Neurobiotin, *in vitro* lucifer yellow, etc.). Each of these attributes is paired with a [Find More] link to assist the user in locating similar models. The metadata also links to the morphology's source (currently specific entries in NeuroMorpho.Org or ModelDB).

[Download Morphology], positioned next to the source link, offers a direct shortcut for downloading just the morphology data. To ensure fidelity to the original reconstruction, this data is shared exactly as entered into our algorithm. No standardization is done for these files; as such, they may be in any of a number of file formats. All we require is that we are able to load the morphology into NEURON; thus each morphology in the database can be both printed and used for simulations.

References associated with a model's morphology are provided at the bottom of the page, along with links to corresponding entries in the journal website, PubMed, NeuroMorpho.Org, or ModelDB, as appropriate. Every neuron in our database is associated with one or more published papers—typically a paper describing the results of neuron tracing experiments or a modeling paper that used the specific reconstruction.

An interactive view of the unmodified 3D morphology is provided in the upper right corner of each model's page. Dragging across this view with the left button pressed on a mouse rotates the image in 3 dimensions, the scroll wheel zooms, and dragging with the right button pressed translates the view position.

One or more printable versions of a cell are listed below the metadata and the morphology viewer. The need for multiple versions arises from the choice of magnification and of compromises (e.g., thickened dendrites) necessary to define a shape that can be printed with existing technologies.

Each printable version lists a name, a comment, the magnification if known, printed volume, bounding box, algorithm that defined the morphology, creator, date added, and a unique id. The printed volume—the amount of material in the final object—and the bounding box are factors in calculating the cost of printing the object with many commercial providers. Finally, each printable version includes a photograph of the printed product with a scale bar (the image enlarges when clicked) and a button for downloading the printable files.

### 3.3. Learning from printed models

**Figure 3:**
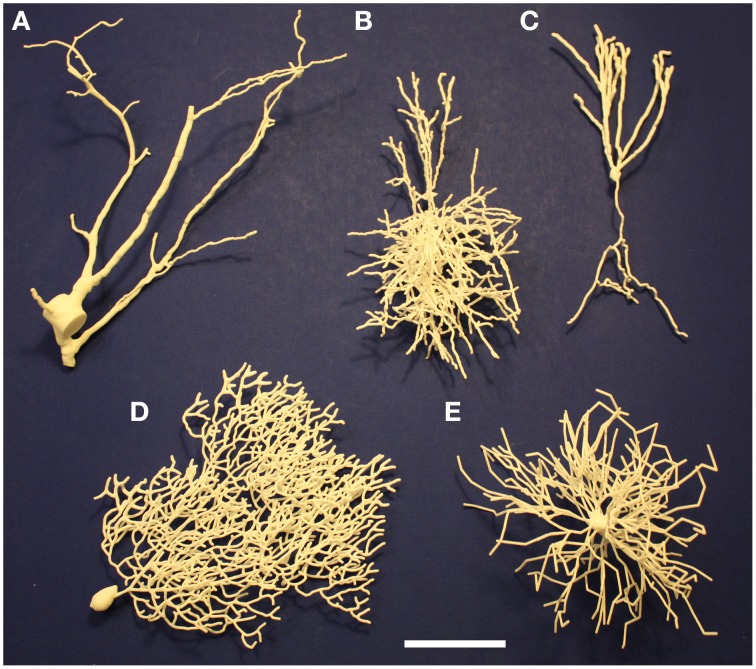
**Examples of 3D-printed neurons. (A)** Spinal cord projection neuron. **(B)** Basket cell. **(C)** Dentate gyrus granule cell. **(D)** Purkinje cell. **(E)** Spinal cord motor neuron. The scale bar is 5 cm.

Examples of 3D printed neurons are shown in Figure 3. All of the neurons in this figure were traced from actual neurons with the exception of the Purkinje cell. Even in these 2D photographic views their 3D quality can be seen.

#### 3.3.1. Single neuron printouts

##### Spinal projection neuron

A lumbar spinal cord projection neuron from the rat, NeuroMorpho.Org's NMO_08992 (Baseer et al., 2012), was printed 800x larger than actual size (Figure 3A). This magnification is sufficient that choosing a fixed dendrite size was not needed, revealing the diversity of dendritic diameters. We can see the relatively large soma, the single very large obviously amputated dendritic trunk, and two other dendrites. The original shape of the soma was not recorded in the SWC source, so it was rendered with a cylinder. At this high magnification, the cylinder approximation of the soma is very noticeable. Nubbins extend from the soma as well as other amputated dendritic trunks and branches. The ability to examine the cell makes clear how it was obtained in a slice preparation, with amputation of most of the dendrites, leaving the three existing dendrites lying in a single plane parallel to the plane of the slices. This is a key factor to take into account in using the morphology to estimate cell properties such as surface area, input resistance, etc.

##### Basket cell L2/3

A basket cell from the upper layers of the rat neocortex (Wang et al., 2002) is shown in Figure 3B. This represents both dendritic and axonal arborizations; to distinguish between them, open the model on 3DModelDB and click the link to NeuroMorpho.Org (C260897C-I1). In their image, it can be seen that the green dendrites form a relatively compact tree around the cell body; the rest of the model therefore is the axonal arborization connecting to the somas of many pyramidal cells in a vertical orientation. Note in both views that one side of the arborization is relatively flat, presumably indicating closeness to one side of the slice preparation from which this cell was taken.

##### Dentate gyrus granule cell

Our printable version of NeuroMorpho.Org's NMO_00154 (Cannon et al., 1998), shows how under favorable circumstances dendrites and axons can be clearly distinguished (Figure 3C; see also **Figure 5A**). In this case the axon was given a diameter of 6 um and the dendrites 9 um. The dendritic arborization is characteristic of dentate granule cells, though somewhat reduced in amount. This was presumably due to loss from a slice, also suggested by the uniplanar morphology, as can be seen immediately while holding the printout. This planarity is also noticeable by viewing the cell in 3DModelDB and rotating it in the box. The dentate granule cell axon becomes the mossy fiber projecting to the CA3 pyramidal cells; initially it gives off collaterals within the dentate gyrus which can be seen clearly in the printout. A limitation of this and all of our printouts is the lack of spines on the dendrites.

##### Cerebellar Purkinje cell

The cerebellar Purkinje cell (Figure 3D) has the most recognizable dendritic tree in the brain, with profuse branching limited to one plane. This is not due to the slicing of the *in vitro* prep; it is the signature of this cell and a key to its function. Parallel fibers from granule cells pass through orthogonal to the plane of the branches, to activate many cells in sequence. Each Purkinje cell has up to 300,000 synapses on its dendritic tree. The synapses are mostly made on dendritic spines, which are not present in the model. An axon (not shown) arises from the cell body. Unlike the other cells in this figure, this neuron's morphology was generated by an algorithm based on statistical properties of traced cells as described in Migliore et al. (2014) instead of traced directly from a living neuron.

##### Spinal motor neuron

In contrast with NMO_08992 in the rat (Figure 3A), the motor neuron of Figure 3E (NeuroMorpho.Org ID NMO_00607; Cullheim et al., 1987)—stained *in vivo* in the cat—shows the motor neuron in its full glory. The eight initial dendritic stems from the soma branch more or less symmetrically to form a luxuriant dendritic tree, though with limited branching on one side. Since this is an *in vivo* stained cell, this may represent a true asymmetry in the branching. The axon is not included in the staining. Analysis of the microscopic images showed 181 branch points and 370 branches as listed in NeuroMorpho.Org; this detailed analysis can obviously be carried out more effectively on the microscopic images than in the fully 3D rendered model, which on the other hand gives a more dramatic impression of the extent of the space filling tree and its slight asymmetry.

Each of these printouts thus enhances the ability of the observer to see similar as well as different aspects of normal neuron morphology, and identify aspects of methodology that impact the morphology that otherwise are concealed and that must be taken into account in interpreting experimental results.

#### 3.3.2. Multiple neuron printouts

We then printed out multiple related neurons as a first step toward printing out neuronal microcircuits. We considered a group of mitral cells connected to a single glomerulus in the olfactory bulb. Since groups of cells are generally not traced together, we used five distinct mitral cells that together form a glomerular cluster in the model of Migliore et al. (2014).

**Figure 4:**
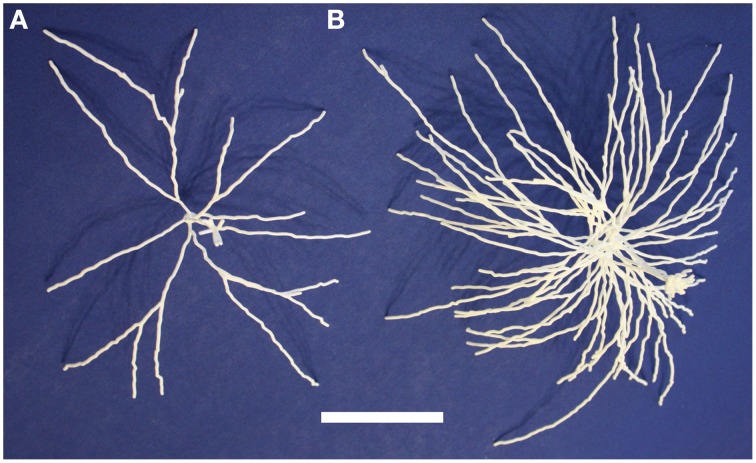
**A single olfactory bulb mitral cell (A) and a cluster of 5 mitral cells (B) from Migliore et al. (2014)**. Printing the cluster in one piece shows the complex interleaving of dendrites of different cells in a microcircuit. The scale bar is 5 cm.

These five neurons are all synthetic neurons obtained by the algorithm explained in that paper's Methods section. Thus, each one of these mitral cells is different from the others, but they are all generated by the same algorithm that ensures that the final parameters of branch length, diameter and angle of origin conform with the experimentally labeled mitral cells of Igarashi et al. (2012). The printout enables one to appreciate more directly the tight convergence of the apical dendrites on a single glomerulus. Note also especially the dense web formed by the lateral dendrites. These properties are the more striking in that the 5:1 ratio of mitral cells to glomeruli shown here is only 1/4 of the normal ratio of 20 to 1. The 3D printouts thus give a new perspective on the network qualities of processing in the nervous system. One of the five mitral cells of the cluster is shown in Figure 4A; the whole cluster is shown in Figure 4B.

## 4. Discussion

Printing neuron reconstructions and artificially generated neuronal morphologies in three-dimensions provides new approaches for understanding the nature and role of these intricate structures. In this article, we have described our technique for generating printable models, the main challenges in applying this technology to neurons, a database to share printable neuron models, and examples of the kinds of insight that can be gained.

The delicate shape of a neuron poses the main technological challenge to constructing these 3D printouts. The dendrites and axons connect at the soma but otherwise do not directly connect and thus do not help each other maintain their shape and position against loads such as gravity. Fluid and the presence of other structures in the brain help actual neurons to retain their shape, but isolated printouts lack these forms of support. Due to the limitations of printer technology, we currently have to print neurites (axons and dendrites) with expanded diameters. This expansion causes branches that pass very close to each other to print physically merged which adds some unnatural structural support. Nonetheless, when we printed the microcircuit model at our thinnest successful thickness, it noticeably flattened under its own weight when placed on a table. The use of virtual reality instead of 3D printing would offer many of the same benefits and allow for realistically tapering dendrites with a relative loss in tactile sensation and in portability.

There have been several use cases suggested by people who have been excited about this technology. One obvious use is in education. Instead of looking at microscopy images that are necessarily two-dimensional projections of neurons, 3D printed neurons allow students to see the full 3D structure, manipulate it, place neurons next to each other to compare structures and see how they overlap. We first used these models for this purpose at Yale's annual Brain Education Day. We passed several printed cells around in small groups to a total of approximately 100 middle and high school students. The printouts were robust enough to survive all the handling, and they were helpful in promoting student excitement about our lesson.

**Figure 5:**
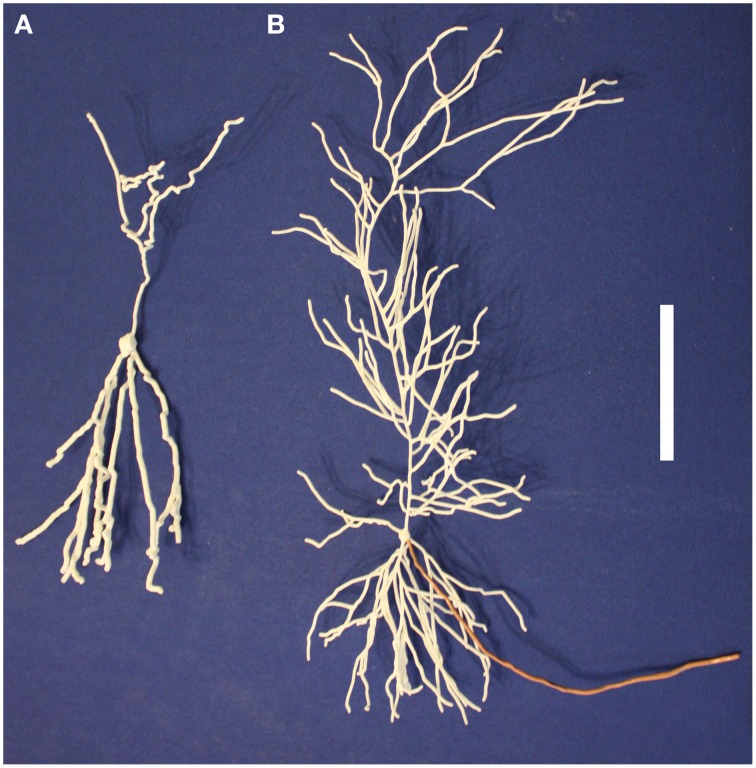
**Neuron regions and properties can be identified on printed models in multiple ways. (A)** The axon (top) of this dentate gyrus granule cell is indicated with a thinner diameter than the dendrites (bottom). **(B)** The axon of this pyramidal neuron has been manually painted to distinguish it from the white dendrites. The scale bar is 5 cm.

3D printing offers a new form of data visualization. Morphologies downloaded from NeuroMorpho.Org or algorithmically grown consist of a collection of points, diameters, and connectivity information. Our technique transforms that data into a physically realizable object. Additional data can be encoded into the printout: neurite diameters can be adjusted to emphasize specific regions (Figure 5A). Colors can be added either as part of the printing process or manually afterward to indicate regions, encode properties (e.g., channel density from a computational model or experimental data), or state (e.g., membrane potential) (Figure 5B). Multiple printouts can be used to indicate the time evolution of states, such as a propagating action potential.

Finally, 3D printing facilitates quality control—both for people who trace neurons and for modelers—by making the morphology data more readily interpretable. Many of the cells that we considered for 3D printing appeared to have been amputated, possibly when the slice was taken. Tracers can use printouts to help identify strategies for improving their technique. Modelers can use them to assess if a morphology offers sufficient realism for their needs.

The single neurons and the glomerular cluster described here are steps toward visualizing 3D neurons in their multi-neuronal context. Connectomics techniques such as automated 3D electron microscopy (Kaynig et al., 2015) are a potentially important future source of high-resolution morphological data for microcircuits. Our longer term goal is to move from showing multiple neurons together to be able to print out microcircuits to visualize how neurons interact.

## Author contributions

RM and GS developed the concept. RM implemented the conversion code and the database. RM and GS wrote the paper.

## Funding

This project was supported by the National Institutes of Health (NIH) grants R01 DC009977 from the National Institute on Deafness and Other Communication Disorders (NIDCD) and T15 LM007056 from the National Library of Medicine (NLM).

### Conflict of interest statement

The authors declare that the research was conducted in the absence of any commercial or financial relationships that could be construed as a potential conflict of interest.

## References

[B1] Al-Kofahi K. A. Lasek S. Szarowski D. H. Pace C. J. Nagy G. Turner J. N.. (2002). Rapid automated three-dimensional tracing of neurons from confocal image stacks. IEEE Trans. Inf. Technol. Biomed. 6, 171–187. 10.1109/TITB.2002.1006304 12075671

[B2] Ascoli G. A. Donohue D. E. Halavi M. (2007). NeuroMorpho.Org: a central resource for neuronal morphologies. J. Neurosci. 27, 9247–9251. 10.1523/JNEUROSCI.2055-07.2007 17728438 PMC6673130

[B3] Baseer N. Polgár E. Watanabe M. Furuta T. Kaneko T. Todd A. J. (2012). Projection neurons in lamina iii of the rat spinal cord are selectively innervated by local dynorphin-containing excitatory neurons. J. Neurosci. 32, 11854–11863. 10.1523/JNEUROSCI.2707-12.2012 22915126 PMC3438856

[B4] Brunjes P. C. Kenerson M. C. (2010). The anterior olfactory nucleus: quantitative study of dendritic morphology. J. Comp. Neurol. 518, 1603–1616. 10.1002/cne.22293 20187150 PMC3546507

[B5] Cannon R. Turner D. Pyapali G. Wheal H. (1998). An on-line archive of reconstructed hippocampal neurons. J. Neurosci. Methods 84, 49–54. 10.1016/S0165-0270(98)00091-0 9821633

[B6] Carey R. Bell G. (1997). The Annotated VRML 2.0 Reference Manual. Essex: Addison-Wesley Longman Ltd.

[B8] Cullheim S. Fleshman J. Glenn L. Burke R. (1987). Membrane area and dendritic structure in type-identified triceps surae alpha motoneurons. J. Comp. Neurol. 255, 68–81. 10.1002/cne.902550106 3819010

[B9] Cuntz H. Forstner F. Borst A. Häusser M. (2010). One rule to grow them all: a general theory of neuronal branching and its practical application. PLoS Comput. Biol. 6:e1000877. 10.1371/journal.pcbi.1000877 20700495 PMC2916857

[B10] Djurisic M. Popovic M. Carnevale N. Zecevic D. (2008). Functional structure of the mitral cell dendritic tuft in the rat olfactory bulb. J. Neurosci. 28, 4057–4068. 10.1523/JNEUROSCI.5296-07.2008 18400905 PMC6670455

[B11] Donohue D. E. Ascoli G. A. (2008). A comparative computer simulation of dendritic morphology. PLoS Comput. Biol. 4:e1000089. 10.1371/journal.pcbi.1000089 18483611 PMC2376061

[B12] Glaser J. R. Glaser E. M. (1990). Neuron imaging with neurolucidaâĂŤ pc-based system for image combining microscopy. Comput. Med. Imaging Graph. 14, 307–317. 10.1016/0895-6111(90)90105-K 2224829

[B13] Hines M. L. Carnevale N. T. (1997). The NEURON simulation environment. Neural Comput. 9, 1179–1209. 10.1162/neco.1997.9.6.1179 9248061

[B14] Hines M. L. Morse T. Migliore M. Carnevale N. T. Shepherd G. M. (2004). ModelDB: a database to support computational neuroscience. J. Comput. Neurosci. 17, 7–11. 10.1023/B:JCNS.0000023869.22017.2e 15218350 PMC3732827

[B15] Igarashi K. M. Ieki N. An M. Yamaguchi Y. Nagayama S. Kobayakawa K.. (2012). Parallel mitral and tufted cell pathways route distinct odor information to different targets in the olfactory cortex. J. Neurosci. 32, 7970–7985. 10.1523/JNEUROSCI.0154-12.2012 22674272 PMC3636718

[B16] Ishizuka N. Cowan W. M. Amaral D. G. (1995). A quantitative analysis of the dendritic organization of pyramidal cells in the rat hippocampus. J. Comp. Neurol. 362, 17–45. 10.1002/cne.903620103 8576427

[B17] Kaynig V. Vazquez-Reina A. Knowles-Barley S. Roberts M. Jones T. R. Kasthuri N.. (2015). Large-scale automatic reconstruction of neuronal processes from electron microscopy images. Med. Image Analysis 22, 77–88. 10.1016/j.media.2015.02.001 25791436 PMC4406409

[B18] Kole M. H. Ilschner S. U. Kampa B. M. Williams S. R. Ruben P. C. Stuart G. J. (2008). Action potential generation requires a high sodium channel density in the axon initial segment. Nat. Neurosci. 11, 178–186. 10.1038/nn2040 18204443

[B19] Kruth J.-P. Wang X. Laoui T. Froyen L. (2003). Lasers and materials in selective laser sintering. Assembly Autom. 23, 357–371. 10.1108/01445150310698652

[B20] Markaki M. Orphanoudakis S. Poirazi P. (2005). Modelling reduced excitability in aged CA1 neurons as a calcium-dependent process. Neurocomputing 65, 305–314. 10.1016/j.neucom.2004.10.023

[B21] McDougal R. A. Hines M. L. Lytton W. W. (2013). Water-tight membranes from neuronal morphology files. J. Neurosci. Methods 220, 167–178. 10.1016/j.jneumeth.2013.09.011 24091136 PMC4197804

[B22] Migliore M. Cavarretta F. Hines M. L. Shepherd G. M. (2014). Distributed organization of a brain microcircuit analyzed by three-dimensional modeling: the olfactory bulb. Front. Comput. Neurosci. 8:50 10.3389/fncom.2014.00050 PMC4010739 24808855

[B23] Nadkarni P. M. Marenco L. Chen R. Skoufos E. Shepherd G. Miller P. (1999). Organization of heterogeneous scientific data using the EAV/CR representation. J. Am. Med. Inform. Assoc. 6, 478–493. 10.1136/jamia.1999.0060478 10579606 PMC61391

[B24] Radman T. Ramos R. L. Brumberg J. C. Bikson M. (2009). Role of cortical cell type and morphology in subthreshold and suprathreshold uniform electric field stimulation *in vitro*. Brain Stimul. 2, 215–228. 10.1016/j.brs.2009.03.007 20161507 PMC2797131

[B25] Ramachandran P. Varoquaux G. (2011). Mayavi: 3D visualization of scientific data. Comput. Sci. Eng. 13, 40–51. 10.1109/MCSE.2011.35

[B26] Tukker J. J. Lasztóczi B. Katona L. Roberts J. D. B. Pissadaki E. K. Dalezios Y.. (2013). Distinct dendritic arborization and *in vivo* firing patterns of parvalbumin-expressing basket cells in the hippocampal area ca3. J. Neurosci. 33, 6809–6825. 10.1523/JNEUROSCI.5052-12.2013 23595740 PMC4473055

[B27] Wang Y. Gupta A. Toledo-Rodriguez M. Wu C. Z. Markram H. (2002). Anatomical, physiological, molecular and circuit properties of nest basket cells in the developing somatosensory cortex. Cereb. Cortex 12, 395–410. 10.1093/cercor/12.4.395 11884355

[B28] Wolf S. Grein S. Queisser G. (2013). Employing NeuGen 2.0 to automatically generate realistic morphologies of hippocampal neurons and neural networks in 3d. Neuroinformatics 11, 137–148. 10.1007/s12021-012-9170-1 23111491

[B29] Zubler F. Douglas R. (2009). A framework for modeling the growth and development of neurons and networks. Front. Comput. Neurosci. 3:25. 10.3389/neuro.10.025.2009 19949465 PMC2784082

